# Tongue sole creatine kinases function as DAMP and activate antimicrobial immunity *via* TLR2

**DOI:** 10.3389/fimmu.2023.1142488

**Published:** 2023-03-01

**Authors:** Xin Li, Shuai Jiang, Li Sun

**Affiliations:** ^1^ CAS and Shandong Province Key Laboratory of Experimental Marine Biology, Institute of Oceanology, CAS Center for Ocean Mega-Science, Chinese Academy of Sciences, Qingdao, China; ^2^ Laboratory for Marine Biology and Biotechnology, Pilot National Laboratory for Marine Science and Technology, Qingdao, China; ^3^ College of Earth and Planetary Sciences, University of Chinese Academy of Sciences, Beijing, China

**Keywords:** creatine kinase, tongue sole, teleost, toll-like receptor 2, antimicrobial immunity

## Abstract

Creatine kinase (CK) is an enzyme that regulates adenosine triphosphate (ATP) metabolism to maintain energy homeostasis. Although CK has been reported to be involved in pathogen infection, the immune function of CK remains elusive. In this study, we identified two muscle-type CK from the teleost tongue sole *Cynoglossus semilaevis* (designated CsCKM-1 and CsCKM-2). Bacterial infection modulated CsCKM-1/2 expression in tongue sole tissues and induced the release of CsCKM-1/2 into serum. Recombinant CsCKM-1/2 (rCsCKM-1/2) exhibited robust kinase activity and bound to bacterial pathogens and pathogen-associated molecular patterns. rCsCKM-1/2 also bound to tongue sole peripheral blood leukocytes (PBLs) and promoted PBLs to uptake bacterial pathogens, inhibit bacterial proliferation, and express proinflammatory cytokines. When co-expressed in HEK293T cells, CsCKM-1/2 were found to interact with the leucine rich domain of toll-like receptor 2 (TLR2). The presence of TLR2 antagonist significantly reduced CsCKM-1/2-induced immune response and antibacterial effect. Taken together, these results indicated that tongue sole creatine kinases function as damage-associated molecular pattern (DAMP) molecules and play an important role in antimicrobial immunity *via* TLR2.

## 1 Introduction

Creatine kinase (CK) is a class of proteins belonging to the phosphagen kinase family (1). CK exists widely in animals, ranging from sponge, fish to human (2). In vertebrates, there are four types of CK, including two cytosolic isoenzymes, i.e., M-CK (muscle type) and B-CK (brain type), and two mitochondrial isoenzymes, i.e., Mi*_u_*-CK (ubiquitous type) and Mi*_s_*-CK (sarcomeric type) (2). The cytosolic type CKs function as homodimer (MM-CK and BB-CK) or heterodimer (MB-CK), while the mitochondrial CKs function as octamer. The CK monomers share similar structure, which is composed of a small N-terminal domain constituted solely by α-helix and a large C-terminal domain comprising an antiparallel β-sheet core and several external α-helices. The two domains form a cleft in their interface and therein accommodate the active site (2–6). CK catalyzes the reversible conversion of phosphocreatine and adenosine diphosphate (ADP) to creatine and adenosine triphosphate (ATP) (4). CK and its substrates/products constitute an energy buffer system enabling replenishment of ATP, the primary fuel of biological activities. Thus, CK plays a vital role in the physiological processes requiring high energy turnover, such as skeletal muscle contraction (2–6).

In human and mouse, accumulating reports indicate that CK is also involved in immunity. For instance, B-CK could modulate thymocyte differentiation and T cell activation (7). The abrogation of B-CK impaired TCR-mediated activation of mTORC1 signaling and compromised CD8^+^ T cell expansion in response to infection (8). The blockade of CK pathway impaired the expression of GSK3-targeted genes and the upstream genes of Wnt signaling (9). B-CK could interact with the NS4A protein of hepatitis C virus (HCV) and affect viral replication (10). Moreover, CK can be released into the extracellular milieu during physiological stress (11–13). In clinical diagnostics, the CK level in body fluid has been widely employed as a biomarker for many pathological conditions, such as exertional muscle damage (14), acute pulmonary tuberculosis (15), acute myocardial infarction (16) and bacterial infection (17, 18).

In teleost, evidence shows that CK is involved in anti-bacterial immunity. For example, in zebrafish *Danio rerio*, the protein level of CK in gill was elevated during *Aeromonas hydrophila* infection (19). In juvenile Nile tilapia *Oreochromis niloticus*, the activities of branchial cytosolic CK and mitochondrial CK decreased upon *Providencia rettgeri* infection (20). In silver catfish *Rhamdia quelen*, the activities of cytosolic and mitochondrial CK were regulated by *Aeromonas caviae* and *Citrobacter freundii* infection (21, 22). In addition, it has been observed that the extracellular level of teleost CK changed upon bacterial infection (23). However, the immunological function and mechanism of CK in teleost remain largely unknown.

Half-smooth tongue sole (*Cynoglossus semilaevis*) is an economically valuable marine flatfish (24). In the present work, we identified and characterized two M-CK homologues from *C. semilaevis* (named CsCKM-1 and CsCKM-2). The immune function and the molecular mechanism of CsCKM-1/2 in antimicrobial immunity were examined. We found that bacterial infection induced extracellular release of CsCKM-1/2, and recombinant CsCKM-1/2 stimulated the activity of peripheral blood leukocytes (PBLs) and exerted antimicrobial effects in a manner that required Toll-like receptor 2 (TLR2). Our results revealed novel immune functions of CK in teleost.

## 2 Materials and methods

### 2.1 Fish, bacteria, and cells

Tongue sole were purchased from a local fish farm in Shandong Province. The fish were reared at 20°C in tanks filled with aerated seawater, fed daily with commercial feed and confirmed to be clinically healthy as described previously (25, 26). Fish with average weight of 500g were used to collect peripheral blood leukocytes (PBLs). Fish with average weight of 20g were used in the bacterial infection experiment. The fish were euthanized with tricaine methane sulfonate (Sigma, St. Louis, USA) at the dose of 0.1 g/l before tissue collection. The bacterial pathogens *Edwardsiella tarda*, *Vibrio anguillarum, Vibrio harveyi* and *Streptococcus iniae* have been reported previously (27–30) *V. anguillarum, V. harveyi* and *E. tarda* were cultured in Luria-Bertani broth (LB) at 28°C to an OD_600_ of 0.8, *S. iniae* was cultured in tryptic soy broth (TSB) at 28°C to an OD_600_ of 0.8. HEK293T cells were cultured in Dulbecco’s modified Eagle’s medium as reported previously (31). Tongue sole peripheral blood leukocytes (PBLs) were prepared by Percoll gradient centrifugation as described previously (32). Briefly, the blood was placed on top of 61% Percoll and centrifuged at 400 g for 15 min. The white layer of PBLs in the interface was collected, washed twice with PBS and resuspended in L-15 medium.

### 2.2 Sequence and phylogenetic analysis

The sequence analysis was performed with Clustal W Multiple Alignment program (33). The phylogenetic tree was constructed using the Maximum likelihood method of MEGA-X with 1500 bootstrap replications, and edited with iTOL (34). The sequences of creatine kinase isoforms were obtained from National Center for Biotechnology Information (NCBI). The GenBank accession numbers are as follows: *Homo sapiens* M-CK, NP_001815. 2; *H. sapiens* B-CK isoform 1, NP_001814.2; *H. sapiens* B-CK isoform 2, NP_001349460.1; *H. sapiens* Mi*_s_*-CK, NP_001093205.1; *H. sapiens* Mi*_u_*-CK, NP_001015001.1; *Oryzias latipes* M-CK, XP_004076079.1; *O. latipes* B-CK, XP_023808469.1; *O. latipes* Mi*_u_*-CK, XP_004069454.3; *O. latipes* Mi*_s_*-CK, XP_023816157.1; *Mus musculus* M-CK, NP_031736.1; *M. musculus* B-CK, NP_067248.1; *M. musculus* Mi*_s_*-CK, NP_940807.1; *M. musculus* Mi*_u_*-CK, NP_001341998.1; *Danio rerio* M-CK b, NP_001099153.1; *D. rerio* M-CK a, NP_571007.2; *D. rerio* B-CK a, NP_001070631.1; *D. rerio* B-CK b, NP_775329.1; *D. rerio* Mi*_s_*-CK, NP_956991.1; *Cynoglossus semilaevis* M-CK-1, XP_008307091.1; *C. semilaevis* M-CK-2, XP_008330730.1.

### 2.3 Expression of CsCKM-1/2 in tongue sole tissues and cells

Expressions of CsCKM-1/2 in tissues under different conditions were determined by quantitative real time reverse transcription-PCR (qRT-PCR). Heart, liver, spleen, head kidney, brain, gill, intestine, skin, and muscle were taken aseptically from three tongue sole for total RNA extraction with Trizol reagent. cDNA synthesis and qRT-PCR were performed as described previously (35) The expression levels of CsCKM-1/2 were analyzed using comparative threshold cycle method (2^-ΔΔCT^). To examine the expression of CsCKM-1/2 in response to infection, tongue sole were randomly divided into three groups and injected intraperitoneally with 50 μl *V. anguillarum* (1 × 10^8^ CFU/ml), *E. tarda* (2 × 10^8^ CFU/ml) or PBS. Three fish were taken at 6, 12, 24 or 48 h post-infection (hpi), and CsCKM-1/2 expressions in liver, spleen and head kidney were determined as above. To examine CsCKM-1/2 expression in PBLs in response to bacterial infection, tongue sole PBLs were incubated with *V. anguillarum* or *E. tarda* (multiplicity of infection (MOI) = 4) for 2, 4, or 6 h, and the expression levels of CsCKM-1/2 were determined by qRT-PCR as above. β-actin was used as an internal reference as reported previously (36). The primers used for PCR are listed in **Table S1**.

### 2.4 Recombinant protein purification, polyclonal antibody preparation, and creatine kinase activity assay

The coding sequences of CsCKM-1/2 were amplified by PCR with primers listed in **Table S1**. The pET30a (+) vector (Novagen, Madison, WI, USA) was digested with the restriction endonucleases NdeI and XhoI. The PCR products were ligated with the linearized vector. The recombinant pET30a (+) vectors expressing CsCKM-1/2 were introduced into *Escherichia coli* Transetta (DE3) (TransGen, Beijing, China) by transformation. The transformants were cultured in LB medium at 37°C to mid-logarithmic phase, and isopropyl-β-D-thiogalactopyranoside was added to the medium to a final concentration of 0.1 mM. After an additional 12 h incubation at 16°C, the bacteria were harvested by centrifugation and lysed on ice by ultrasonication. The recombinant CsCKM-1/2 (rCsCKM-1/2) in the supernatant were purified with Ni-NTA agarose (QIAGEN, Valencia, USA). Triton X-114 was added to the washing buffer to remove endotoxin. The endotoxin levels of the recombinant proteins were determined using Toxin SensorTM Endotoxin Detection System (GenScript). The endotoxin concentrations of rCsCKM-1&2 were 0.24 and 0.17 EU/mg, respectively. The eluted proteins were dialyzed and concentrated as described previously (30). The proteins were analyzed by SDS-PAGE and stained with Coomassie brilliant blue R-250. Mouse anti-rCsCKM-1/2 antiserum was prepared as reported previously (37). Recombinant Trx was prepared as described previously (38). The enzyme activity of recombinant CKs was measured as reported previously (6). Briefly, substrate chromogenic solution was prepared as follows: creatine (25mM), MgAC_2_·4H_2_O (5mM), thymol blue (0.01%), Gly-NaOH (pH 9.0, 5mM), and ATP·Na_2_ (5mM). NaOH (1 mol/L) was used to carefully adjust the color of the solution to dark purple (pH 8.5). rCsCKM-1/2 was then added to the chromogenic solution and the absorption at 597nm was recorded every 30s.

### 2.5 The *in vivo* and *in vitro* detection of CsCKM-1/2 release

To examine the release of CsCKM-1/2 into serum, tongue sole (average 20 g) were randomly divided into three groups and injected intraperitoneally with 50 μl *V. anguillarum* (1 × 10^8^ CFU/ml), *E. tarda* (2 × 10^8^ CFU/ml) or PBS. Blood was collected at 6, 12 and 24 hpi (three fish per group at each time point), and the serum was separated by centrifugation at 2000 g, 4°C for 15 minutes. The serum was subjected to SDS-PAGE, and the proteins were transferred onto nitrocellulose membranes (Millipore, MA, USA). The membrane was blocked with 5% BSA for 1 h, and mouse anti-rCsCKM-1/2 antiserum (1:1000 dilution) were added. After incubation for 1 h, the membranes were washed with PBS for three times. The membranes were further incubated with HRP goat anti-mouse IgG (1:4000 dilution) for 1h, and then washed as above. The protein bands were visualized using an ECL kit (Sparkjade Biotechnology Co. Ltd., Shandong, China). To examine the release of CsCKM-1/2 to culture medium, PBLs (1 × 10^7^ cells/ml) were incubated with *V. anguillarum* (2 × 10^7^ CFU/ml)*, E. tarda* (1 × 10^7^ CFU/ml), or PBS for 1 h. The supernatants were collected and precipitated using TCA as reported previously (31). The proteins released by PBLs were detected by immunoblotting as above.

### 2.6 Interaction of rCsCKM-1/2 with bacteria

The binding between bacteria and rCsCKM-1/2 was determined by enzyme-linked immunosorbent assay (ELISA) as reported previously (39). The binding of rCsCKM-1/2 to pathogen-associated molecular patterns (PAMPs), including lipopolysaccharides, peptidoglycan, glucan and mannan, was examined as follows. PAMPs (0.1 mg/ml) were diluted in coating buffer (15 mM Na_2_CO_3_ and 35 mM NaHCO_3_, pH 9.6) and placed into 96-well microtiter ELISA plates. The plates were incubated at 4°C overnight. After blocking with 5% skim milk at 37°C for 1 h, the plates were washed with PBS for three times. The plates were incubated with different concentrations (1, 5, or 10 μM) of rCsCKM-1/2, followed by incubating with mouse anti-His tag antibody and HRP-conjugated goat anti-mouse IgG as described above. To examine the effect of rCsCKM-1/2 on bacterial growth, rCsCKM-1/2 (85 μg/ml), rTrx (85 μg/ml) or PBS was mixed with bacteria (1 × 10^5^ CFU/ml) in LB medium. The bacteria were incubated at 22°C, and bacterial growth was recorded every hour by measuring absorbance at OD_600_.

### 2.7 The interaction of rCsCKM-1/2 with PBLs

The interaction between rCsCKM-1/2 and PBLs were performed as reported previously (35). Briefly, 1 ×10^6^ cells in 100 μl PBS were settled on the cell culture dish (NEST Biotechnology, Wuxi, China). The dish was blocked with 5% BSA for 1 h and washed with PBS for three times. Then cells were incubated with rCsCKM-1/2 (42.5 μg/ml) or rTrx (42.5 μg/ml) for 1 h and washed as above. Mouse anti-His tag antibody (1:1000 dilution) was added to the dish, and the dish was incubated for 1 h and washed. Goat anti-mouse IgG Alexa Flour 488 (1:2000 dilution) was added to the dish, and the dish was incubated and washed as above. The cells were stained with DAPI and observed with a confocal microscope (Carl Zeiss, Oberkochen, Germany).

### 2.8 The effect of rCsCKM-1/2 on immune gene expression and bacterial infection

Tongue sole were divided randomly into four groups and injected intraperitoneally with 50 μl rCsCKM-1/2 (85 μg/ml), rTrx (85 μg/ml) or PBS. At 6 h post-injection, liver and spleen were taken (three fish per group at each time point) for RNA extraction and cDNA synthesis as described above. cytokine and antimicrobial peptide expression was determined by qRT-PCR with primers listed in **Table S1**. β-actin was used as the internal reference. To examine cytokine expression in PBLs, tongue sole PBLs were incubated with rCsCKM-1/2 (85 μg/ml), rTrx (85 μg/ml) or PBS for 1, 3 and 6 h. RNA extraction and cDNA synthesis were performed as above, and the expression of cytokines and antimicrobial peptides was determined by qRT-PCR as above. To examine the effect of rCsCKM-1/2 on the antimicrobial activity of PBLs, PBLs were pretreated with rCsCKM-1/2 (85 μg/ml), rTrx (85 μg/ml) or PBS for 2 h, followed by incubation with *E. tarda* (MOI=4) for 2 h. Extracellular bacteria were killed by gentamicin (400 μg/ml) for 30 minutes. The cells were placed in fresh medium (this time point was set as 0 h) and incubated for 2 and 4 h. The cells were counted and lysed as reported previously (35). The lysate was diluted and plated on LB agar plates. After incubation at 28°C for 48 h, the colony number was recorded.

### 2.9 The effect of TLR2 antagonist on CsCKM-1/2-induced immune response

Tongue sole PBLs were pretreated with DMSO (control) or different concentrations (1 and 10 μM) of CU-CPT22 (TLR2 specific antagonist) (Selleck.cn, Houston, TX, USA) for 2 h, followed by incubation with rCsCKM-1/2 (85 μg/ml), rTrx (85 μg/ml) or PBS for 1 h. After incubation, the expression levels of IL-1β, IL-6 and TNF-α were determined by qRT-PCR as above. To examine the effect of CU-CPT22 on phagocytosis, tongue sole PBLs in L-15 medium were settled on 24-well plates (Corning Incorporated, Costar) and treated with CU-CPT22 (10 μM) or DMSO (control) for 2 h. The cells were then incubated with rCsCKM-1/2 (85 μg/ml), rTrx (85 μg/ml) or PBS for 2 h as above. *E. tarda* (MOI=4) was added and incubated for another 2 h. After killing the extracellular bacteria, the number of intracellular bacteria was counted by spread plate method as described above.

### 2.10 The interaction between CsCKM-1/2 and TLR2

Confocal microscopy and co-immunoprecipitation were performed to examine the interaction between CsCKM-1/2 and TLR2. For confocal microscopy, recombinant pmCherry-N1 vectors expressing CsCKM-1/2 were constructed as follows. The coding sequences of CsCKM-1/2 were amplified by PCR. The pmCherry-N1 vector was digested with the restriction endonucleases HindIII and BamHI. The PCR products were ligated with the linearized vector. The recombinant vectors were introduced into *E. coli* Trelief 5α (Tsingke Biological Technology, Beijing, China) by transformation. The pEGFP-C1 vector expressing TLR2 leucine rich domain (LRR) was constructed as follows. The coding sequence of TLR2-LRR was amplified by PCR and inserted into the pEGFP-C1 vector at between the HindIII and BamHI sites. HEK293T cells were transfected with vectors expressing mCherry-tagged CsCKM-1/2 or GFP-tagged TLR2-LRR alone, or co-transfected mCherry-tagged CsCKM-1/2 with vector expressing GFP-tagged TLR2-LRR using PolyJet Transfection Reagent (SignaGen, USA). At 24 h after transfection, the cells were observed with a confocal microscope as above. For co-immunoprecipitation, pCS2-6Myc vectors expressing CsCKM-1/2 and TLR2-LRR were constructed as follows. The coding sequences of CsCKM-1/2 and TLR2-LRR were amplified by PCR. The pCS2-6Myc vector was digested with the restriction endonucleases EcoRI and XbaI. The PCR products were ligated with the linearized vector. The pCMV-N-Flag vectors expressing CsCKM-1/2 and TLR2-LRR were constructed as follows: the coding sequences of CsCKM-1/2 and TLR2-LRR were amplified by PCR. The pCMV-N-Flag vector was digested with the restriction endonucleases EcoRI and NheI. The PCR products were ligated with the linearized vector. HEK293T cells were transfected with Flag-tagged CsCKM-1/2 and Myc-tagged TLR2-LRR, or Myc-tagged CsCKM-1/2 and Flag-tagged TLR2-LRR for 24 h. The cells were lysed and incubated with pretreated anti-Flag affinity gel (Beyotime, Shanghai) at 4°C overnight and washed with PBS for seven times. The samples were boiled and used for immunoblotting with HRP-conjugated mouse anti-Myc-tag IgG and HRP-conjugated mouse anti-Flag-tag IgG. The blot was visualized using the ECL kit. All the PCR primers used are listed in **Table S1**.

### 2.11 Statistical analysis

All statistical analysis were carried out with GraphPad Prism 8.4.0 (GraphPad Software, La Jolla, CA, USA). The significance between groups was analyzed with student’s t-test, and statistical significance was defined as P < 0.05.

## 3 Results

### 3.1 Tongue sole possesses two M-CKs with phosphotransferase activity

**Figure 1:**
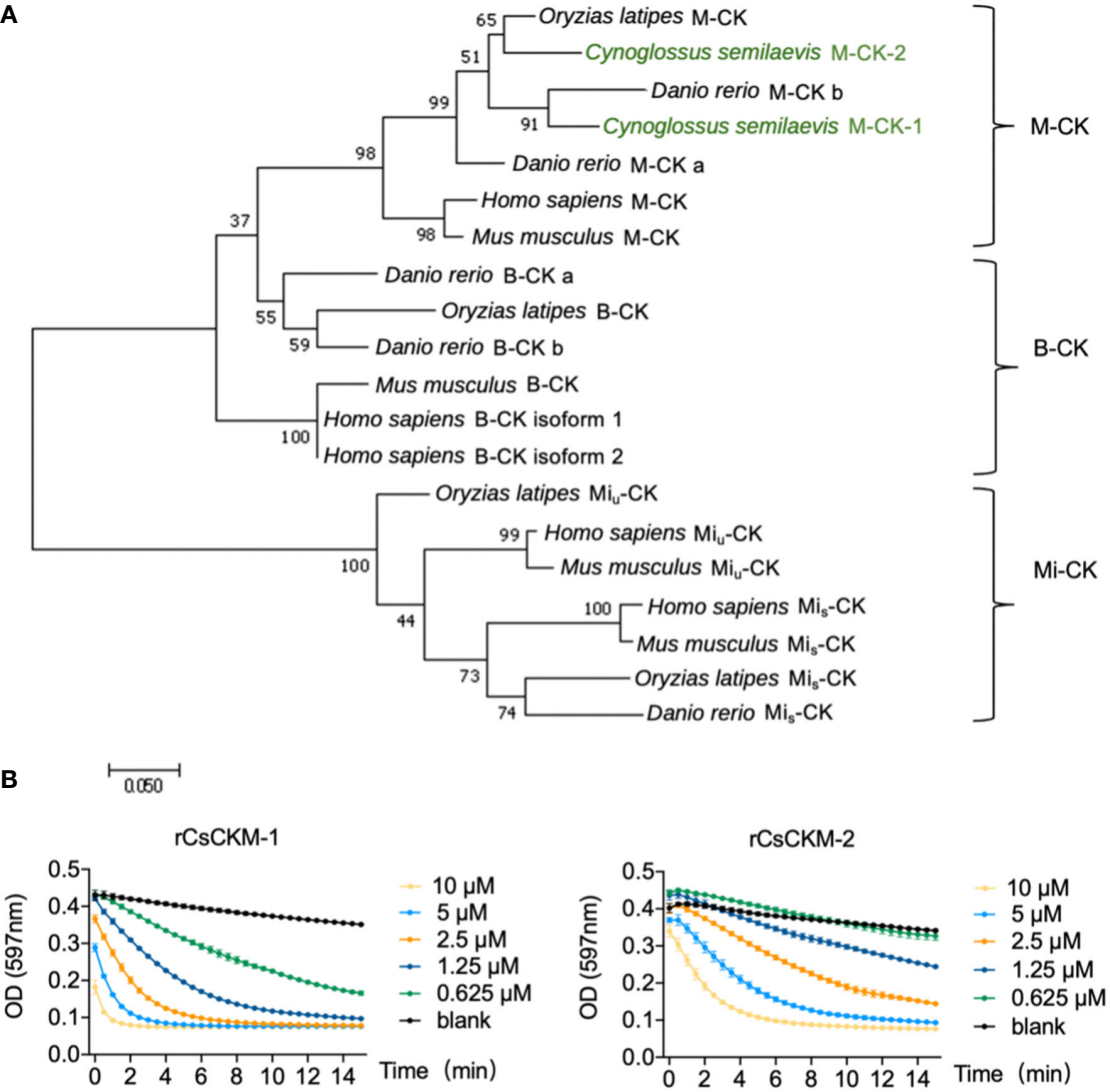
Phylogenetic and activity analyses of CsCKM-1/2. **(A)** Phylogenetic analysis of CK homologs. The numbers indicate bootstrap values based on 1500 replications. M-CK, muscle type CK; B-CK, brain type CK; Mi-CK, mitochondrial type CK; Mi*_u_*-CK, ubiquitous type Mi-CK; Mi*_s_*-CK, sarcomeric type Mi-CK. **(B)** rCsCKM-1/2 at different concentrations were incubated with the substrates. The enzymatic reaction was recorded every 30 seconds by monitoring the absorbance of thymol blue (pH indicator) at 597 nm, because creatine kinase catalysis generates hydrogen ions.

By searching the genomic database, we identified two creatine kinases (CKs) from tongue sole *C. semilaevis*. The two CKs share high sequence identity with each other, and share 85.4% and 85.8% sequence identities with human M-CK, respectively (**Figure S1**). Phylogenetic analysis showed that the tongue sole CKs were phylogenetically grouped together with teleost (*Danio rerio* and *Oryzias latipes*) and human the M-CKs (**Figure 1A**). Based on these results, the two tongue sole CKs were named muscle type CKs (CsCKM-1/2). To examine the activity of CsCKM-1/2, recombinant CsCKM-1/2 (rCsCKM-1/2) were prepared (**Figure S2**). rCsCKM-1/2 exhibited robust phosphotransferase activity in a manner that positively correlated with the concentration of rCsCKM-1/2 (**Figure 1B**).

### 3.2 Bacterial infection stimulates the expression and extracellular release of CsCKM-1/2

**Figure 2:**
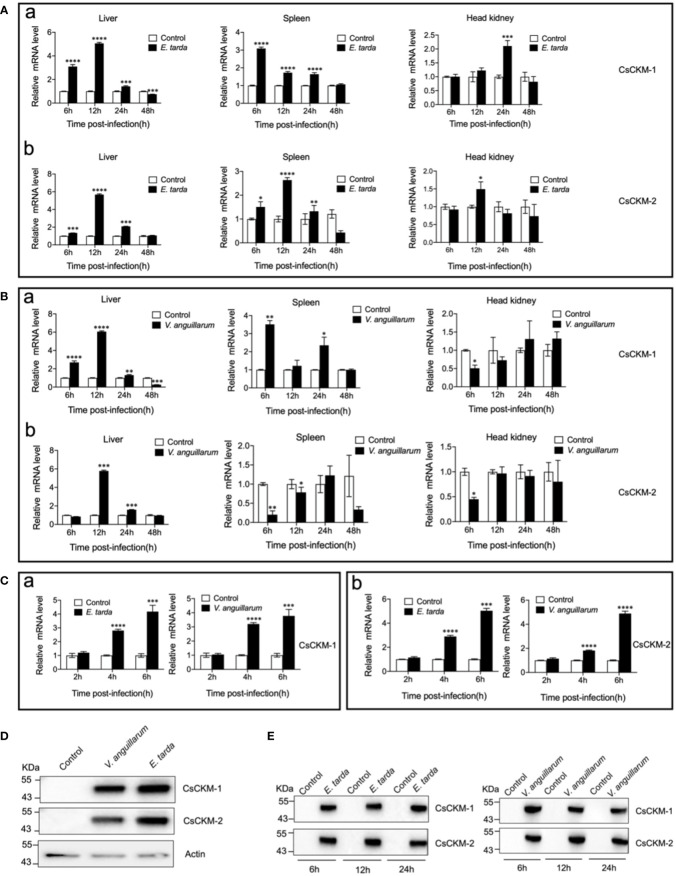
The effect of bacterial infection on CsCKM-1/2 expression and extracellular release. **(A, B)** Tongue sole were infected with or without (control) *Edwardsiella tarda* **(A)** or *Vibrio anguillarum* **(B)**, and the expression levels of CsCKM-1 (a) and CsCKM-2 (b) in liver, spleen and head kidney were determined by qRT-PCR at various time points. **(C)** Tongue sole PBLs were infected with or without (control) *E. tarda* or *V. anguillarum*, and the expression of CsCKM-1(a) and CsCKM-2 (b) was determined by qRT-PCR. In all panels, the expression level in the control fish/cells was set as 1. Values are the means of triplicate assays and shown as means ± SD. *P < 0.05; **P < 0.01; ***P < 0.001; ****P < 0.0001. **(D)** Tongue sole PBLs were infected with or without (control) *E. tarda* or *V. anguillarum*. Extracellular release of CsCKM-1/2 was detected by immunoblotting using anti-CsCKM-1/2 antibodies. β-actin was used as a loading control. **(E)** Tongue sole were infected with or without (control) *E. tarda* or *V. anguillarum* for 6 to 24 h. CsCKM-1/2 in serum were determined by immunoblotting as above.

Under normal physiological condition, CsCKM-1 expression was detected in muscle, skin, heart, liver, head kidney, brain, spleen, gill and intestine (**Figure S3**). The expression pattern of CsCKM-2 was largely similar to that of CsCKM-1. When tongue sole were infected with *Edwardsiella tarda*, the expression of CsCKM-1/2 significantly increased at 6 to 24 hpi in liver and spleen, and at 12 or 24 hpi in head kidney (**Figure 2A**). When the fish were infected with *Vibrio anguillarum*, CsCKM-1 expression significantly increased in liver (6 to 48 hpi) and spleen (6 and 24 hpi), but decreased in head kidney at 6 hpi; CsCKM-2 expression significantly increased in liver (12 and 24 hpi) and decreased in spleen (6 and 12 hpi) and head kidney (6 hpi) (**Figure 2B**). In tongue sole PBLs, both *E. tarda* and *V. anguillarum* infections significantly enhanced CsCKM-1/2 expression at 4 and 6 hpi (**Figure 2C**). Furthermore, *E. tarda* and *V. anguillarum* infections caused extracellular production of CsCKM-1/2 (**Figure 2D**). Consistently, *in vivo* study showed that both *E. tarda* and *V. anguillarum* infections induced secretion of CsCKM-1/2 into tongue sole serum (**Figure 2E**).

### 3.3 CsCKM-1/2 bind bacterial pathogens and pathogen-associated molecular patterns (PAMPs)

**Figure 3:**
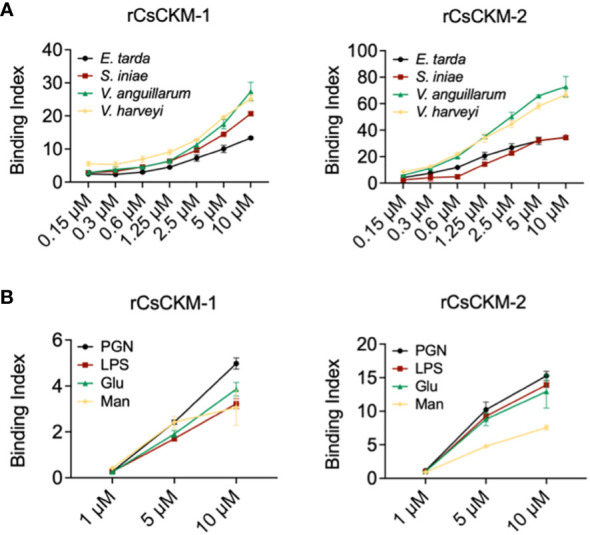
Binding of CsCKM-1/2 to bacteria and bacterial cell surface components. **(A)** rCsCKM-1/2 in different concentrations were incubated with *Edwardsiella tarda*, *Vibrio anguillarum*, *Vibrio harveyi* and *Streptococcus iniae*, and the binding was determined by ELISA. **(B)** The binding of rCsCKM-1/2 to different pathogen-associated molecular patterns (PAMPs) was determined by ELISA. LPS, lipopolysaccharides; PGN, peptidoglycan; Glu, glucan; Man, mannan. Values are the means of triplicate assays and shown as means ± SD.

To examine whether bacteria-induced production of CsCKM-1/2 had any role in immune defense against bacterial pathogens, we determined the potential of CsCKM-1/2 to interact with bacteria. The results showed that rCsCKM-1/2 exhibited dose-dependent binding to the fish bacterial pathogens *E. tarda*, *V. anguillarum*, *Vibrio harveyi* and *Streptococcus iniae* (**Figure 3A**). rCsCKM-1/2 also exhibited strong binding to PAMPs, including lipopolysaccharides (LPS), peptidoglycan (PGN), glucan (Glu) and mannan (Man) (**Figure 3B**). rCsCKM-1/2 had no apparent effect on the growth of *E. tarda*, *V. anguillarum*, *V. harveyi* and *S. iniae* (**Figure S4**).

### 3.4 CsCKM-1/2 promote proinflammatory cytokine expression and reduce bacterial infection

**Figure 4:**
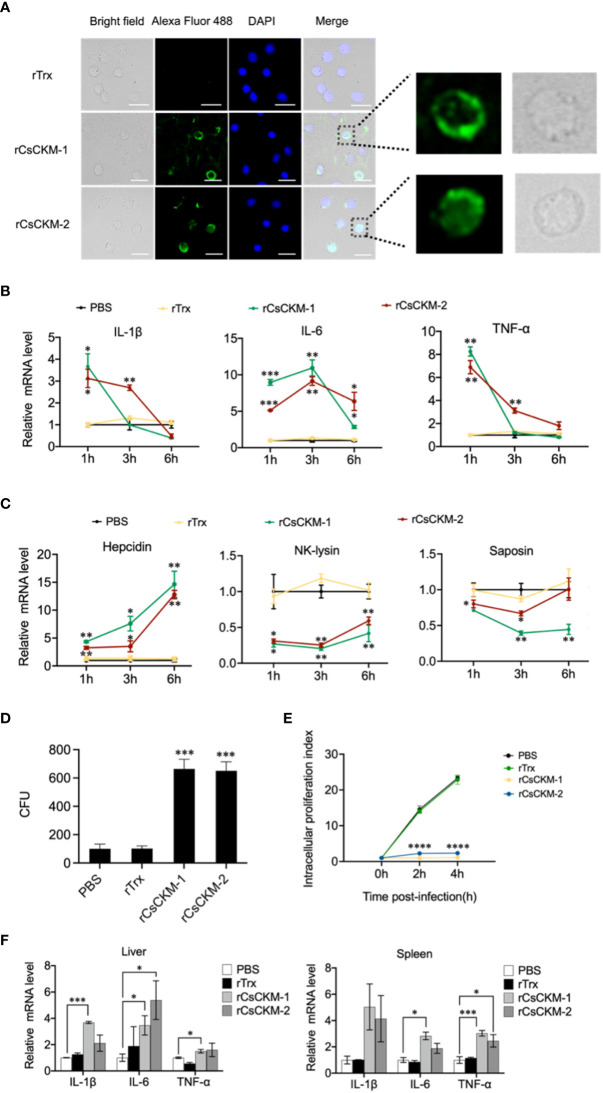
The effect of CsCKM-1/2 on IL-1β, IL-6 and TNF-α expression and *Edwardsiella tarda* infection. **(A)** His-tagged rCsCKM-1/2 or rTrx was incubated with tongue sole PBLs for 1 h. The cells were stained with DAPI and treated with anti-His tag antibody and Alexa Fluor 488-labeled secondary antibody. The distribution of rCsCKM-1/2 in one cell was shown in the enlarged images. Scale bar, 10 μm. **(B)** The expression of IL-1β, IL-6 and TNF-α in PBLs treated with rCsCKM-1/2, rTrx, or PBS (control) was determined at different times by qRT-PCR. **(C)** The expression of hepcidin, NK-lysin and saposin in PBLs treated with rCsCKM-1/2, rTrx, or PBS (control) was determined as above. **(D)** PBLs were pretreated with rCsCKM-1/2, rTrx, or PBS (control) for 2 h, and then incubated with *E. tarda* for 2 h. The number of phagocytosed bacteria was determined. **(E)** PBLs were pretreated with rCsCKM-1/2, rTrx, or PBS (control) and then infected with *E. tarda*. Intracellular bacteria replication at different hours was determined. **(F)** Tongue sole were infected with rCsCKM-1/2, rTrx, or PBS (control), and the expression of IL-1β, IL-6 and TNF-α in spleen and liver was determined by qRT-PCR. For **(B–D)**, values are the means of triplicate assays and shown as means ± SD. *P < 0.05; **P < 0.01; ***P < 0.001, ****P < 0.001.

Since CsCKM-1/2 were released by PBLs during bacterial infection, we examined whether CsCKM-1/2 were able to interact with PBLs. The result showed that following incubation with PBLs, rCsCKM-1/2 were found to bind to PBLs (**Figure 4A**) and significantly upregulated the expression of IL-1β, IL-6 and TNF-α (**Figure 4B**). IL-1β and TNF-α were significantly induced by rCsCKM-1 at 1 hpi and by rCsCKM-2 at 1 and 3 hpi; IL-6 was significantly induced by rCsCKM-1/2 at 1 to 6 hpi. In addition, we found that both CsCKM-1 and CsCKM-2 significantly up-regulated the expression of hepcidin and down-regulated the expression of NK-lysin and saposin (**Figure 4C**). Compared to control cells, rCsCKM-1/2-treated PBLs exhibited enhanced ability to uptake *E. tarda* and inhibit the intracellular replication of *E. tarda* (**Figures 4D, E**). *In vivo* study showed that in tongue sole, rCsCKM-1 significantly upregulated IL-6 and TNF-α expression in liver and spleen, and significantly upregulated IL-1β expression in liver; rCsCKM-2 significantly upregulated IL-6 expression in liver and TNF-α expression in spleen (**Figure 4F**).

### 3.5 TLR2 is required for CsCKM-1/2-mediated immune response

**Figure 5:**
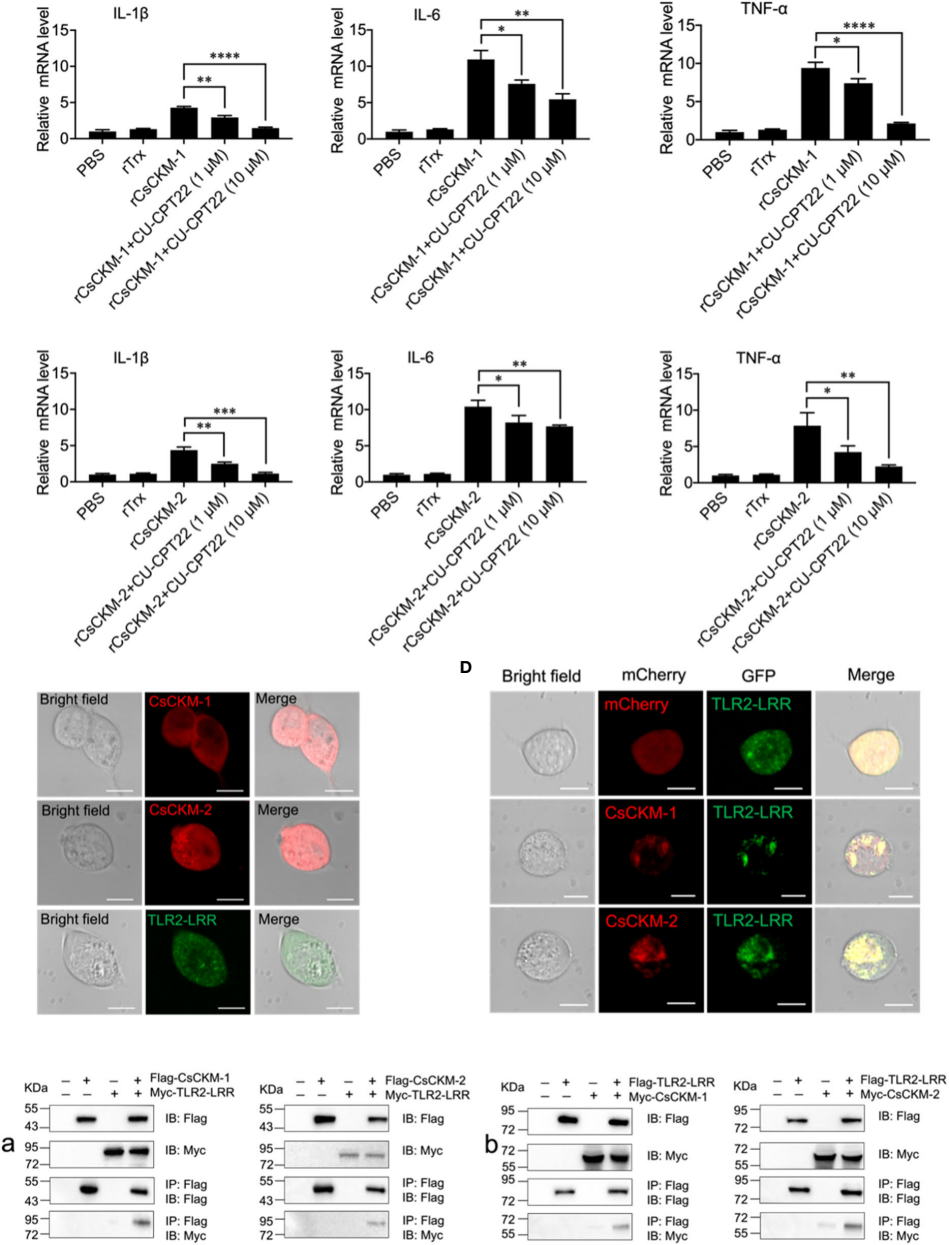
The involvement of TLR2 in CsCKM-1/2-induced immune response. **(A)** Tongue sole PBLs were incubated with PBS, rTrX, rCsCKM-1, or rCsCKM-1 plus different concentrations of CU-CPT22 for 2 h. The expression of IL-1β, IL-6 and TNF-α was determined by qRT-PCR. **(B)** PBLs were incubated with PBS, rTrX, rCsCKM-2, or rCsCKM-2 plus different concentrations of CU-CPT22 for 2 h. Gene expression was determined as above. For both **(A, B)**, values are the means of triplicate assays and shown as means ± SD. *P < 0.05; **P < 0.01; ***P < 0.001, ****P < 0.0001. **(C)** HEK293T cells were transfected with mCherry-tagged CsCKM-1/2 or GFP-tagged TLR2-LRR and observed with a confocal microscope. **(D)** HEK293T cells co-expressed GFP-tagged TLR2-LRR and the mCherry tag, or co-expressed TLR2-LRR and mCherry-tagged CsCKM-1/2. Protein colocalization was observed with a confocal microscope. Scale bar, 10 μm. **(E)** Flag-tagged CsCKM-1/2 and Myc-tagged TLR2-LRR (Ea), or Myc-tagged CsCKM-1/2 and Flag-tagged TLR2-LRR (Eb) were co-expressed in HEK293T cells for 24 h. Co-immunoprecipitation and immunoblotting were performed with antibodies against Myc-tag and Flag-tag.

In order to explore the molecular mechanism of CsCKM-1/2-induced immune response, we investigated whether TLR2 was involved in CsCKM-1/2-induced cytokine expression. The result showed that when PBLs were pretreated with the TLR2 antagonist CU-CPT22, which inhibited TLR2 signaling (40), rCsCKM-1/2-induced expression of IL-1β, IL-6 and TNF-α was significantly suppressed (**Figures 5A, B**). Similarly, the boosting effect of rCsCKM-1/2 on phagocytosis was abolished (**Figure S5**). When expressed in HEK293T cells, CsCKM-1/2 and TLR2 leucine-rich domain (LRR) were evenly distributed in the cytoplasm (**Figure 5C**). When CsCKM-1/2 were co-expressed with TLR2-LRR, co-localization of CsCKM-1/2 with TLR2-LRR was detected by microscopy (**Figure 5D**). Consistently, CsCKM-1/2 were co-immunoprecipitated with TLR2-LRR (**Figure 5E**), suggesting interaction between CsCKM-1/2 and TLR2-LRR.

## 4 Discussion

CK exists in all vertebrates and reversibly catalyzes the reaction of ATP production and consumption (2, 41). Different CK isoenzymes have distinct patterns of tissue specific expression and subcellular localization, which is important to maintain energy homeostasis (42, 43). In teleost, the transcription of CK can respond to various stimulations including temperature, salinity, muscle damage and bacterial infection (19, 44–46). The activity and content of intracellular creatine kinase vary upon bacterial infection in fish such as zebrafish, rainbow trout and Nile tilapia (19, 20, 23, 47). In this study, we found that the two muscle type CKs from tongue sole *C. semilaevis* share over 80% identities with human M-CK, suggesting conserved function of CK in vertebrates. Consistently, rCsCKM-1/2 exhibited robust kinase activity. CsCKM-1/2 expression was detected at high levels in muscle, where CK may provide support for energy homeostasis. CsCKM-1/2 also expressed in immune organs, including spleen and liver, in which the expression of CsCKM-1/2 was significantly elevated after *E. tarda* or *V. anguillarum* infection, suggesting involvement of CK in fish antibacterial immunity.

Under normal physiological conditions, CKs are mainly distributed in the cytoplasm, whereas under pathological conditions, they are released into the extracellular environment. Hence, CKs have been widely used as biomarkers in the early diagnosis of various diseases, including myocardial infarction, Alzheimer’s Disease and seizure disorders (13, 48, 49). In human, bacterial infection could induce the release of CK into serum (17). Similar results were observed in teleost. In rainbow trout, CK was the most upregulated enzyme in the serum during *Aeromonas salmonicida* infection (50). However, the function of CK in serum remains elusive. In mammals, it has been proposed that extracellular CK could modulate purinergic signaling, which is associated with the antimicrobial mechanisms of host immune cells (51, 52). In amphioxus *Branchiostoma belcheri*, CK was able to bind *E. coli* and inhibit bacterial growth through a mechanism similar to that of lectin (53). In speckled hind (*Epinephelus drummondhayi*), the branchial muscle-type CK could interact with the envelope proteins of *Edwardsiella tarda* (54). In our study, we found that under the normal conditions, CsCKM-1/2 could not be detected in serum. However, during the infection of *E. tarda* or *V. anguillarum*, CsCKM-1/2 were detected in serum, indicating bacteria-induced release of CsCKM-1/2. rCsCKM-1/2 exhibited apparent binding capacity to fish pathogens, including *E. tarda*, *V. anguillarum, V. harveyi* and *S. iniae*, probably through interaction with microbial surface PAMPs, such as LPS and PGN. rCsCKM-1/2 lacked bactericidal activity but enhanced the phagocytosis of PBLs, suggesting that extracellular CsCKM-1/2 likely function to promote the antimicrobial activity of PBLs. In addition, as the phosphagen kinases that regulate ATP/ADP homeostasis, the released serum CsCKs might also exert immune effects *via* ATP. Previous studies reported that there existed conserved extracellular ATP-activated signaling pathways in fish, which activated innate immunity and induced cell apoptosis, whereas the hydrolytic product of extracellular ATP, adenosine, exhibited inhibitory effect on the immunity (55, 56). It is possible that serum CsCKs might modulate tongue sole immunity *via* the regulation of the purinergic signaling pathway.

In addition to binding bacteria, CsCKM-1/2 also bound PBLs and upregulated the expression of IL-1β, IL-6 and TNF-α. Similarly, *in vivo* administration of CsCKM-1/2 increased the expression of these cytokines in tongue sole tissues. These results indicated that CsCKM-1/2 probably function as damage-associated molecular patterns (DAMPs) that were released into the extracellular environment in response to bacterial infection and acted as a warning signal to the immune system. Previous studies showed that HMGB1, a classical and widely studied DAMP molecule, mediates sepsis by transferring LPS to CD14, which facilitates the activation of TLR4 (57). In the present study, we found that PBLs pretreated with rCsCKM-1/2 exhibited enhanced phagocytosis and inhibition of the intracellular proliferation of *E. tarda*, indicating an immune activation capacity of CsCKM-1/2.

Host immune response depends on various receptors to recognize extracellular signals, and the evolutionarily conserved pattern recognition receptors (PRRs) play important roles in this process (58). Previous studies showed that certain PRRs (e.g., TLRs) can be activated by DAMPs (59) and initiate downstream signaling pathways, such as the NF-κB, MAPK, cGAS and RIG-I signaling pathways, which result in cytokine production and immune activation (59–61). In human, TLR2/MAPKs signaling pathway is crucial for microbicidal phagocytosis (62). In tongue sole, cell membrane TLR2 was reported to play an important role in cytokine expression and phagocytosis of bacteria (24). In our present study, we found that inhibition of TLR2 signaling significantly decreased CsCKM-1/2-induced proinflammatory cytokine expression and bacterial uptake, indicating involvement of TLR2 in CsCKM-1/2-induced immune response. Consistently, CsCKM-1/2 were found to be co-localized with TLR2-LRR and were co-immunoprecipitated with TLR2-LRR, suggesting that TLR2 likely serves as a receptor for CsCKM-1/2 as DAMPs. The findings collectively suggested a possibility that the bacterial binding ability of CsCKs may facilitate CsCKs to effectively target the bacteria-bound immune cells and interact with the cell surface TLR2, leading to enhanced phagocytosis and other immune response.

**Figure 6:**
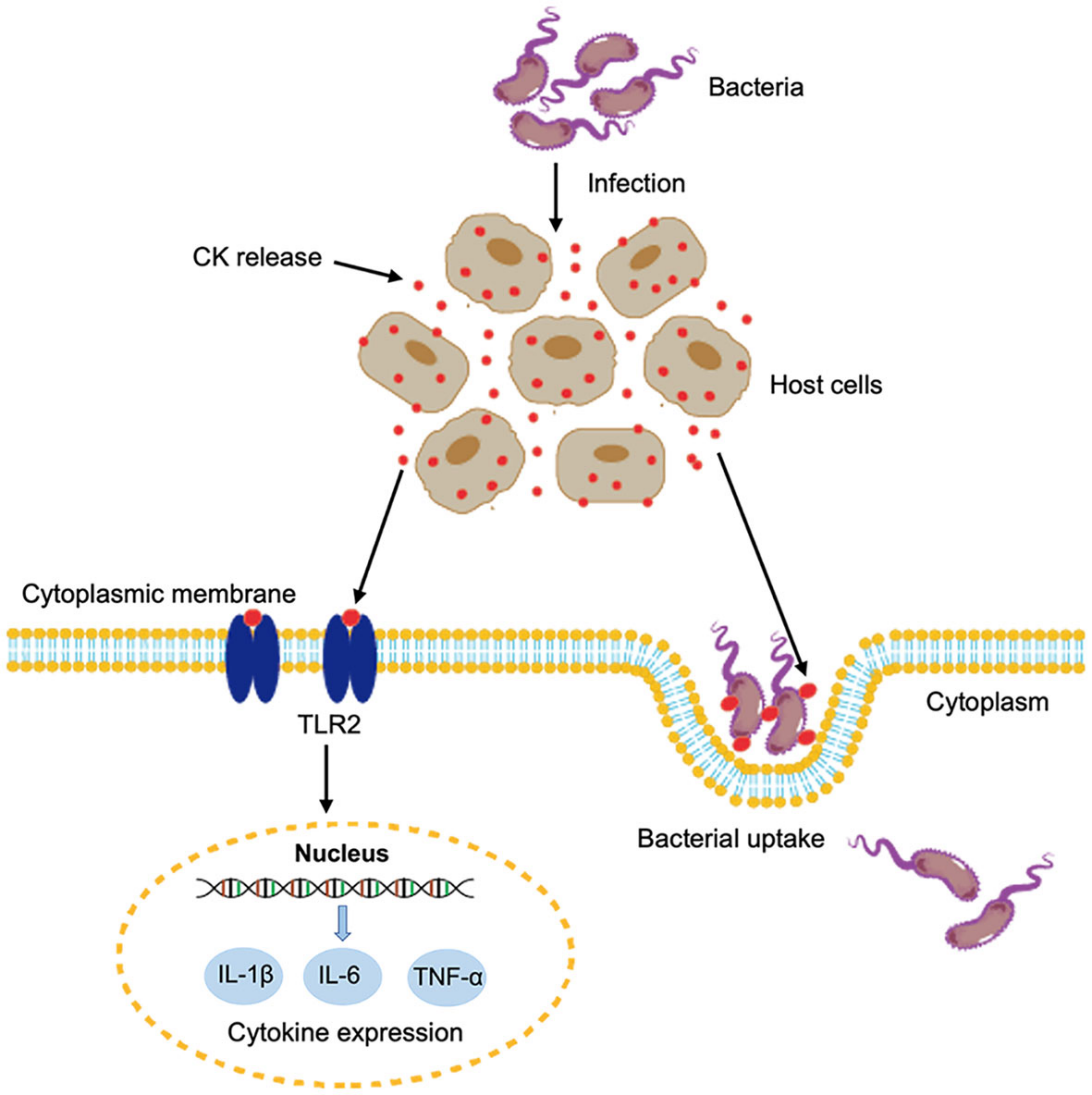
A proposed model of the immune function of CsCKM-1/2. Bacterial infection induces the release of CsCKM-1/2. CsCKM-1/2 interact with TLR2 and activate TLR2-mediated signaling, which induces cytokine expression and likely inhibits bacterial infection. In addition, CsCKM-1/2 bind to the bacterial pathogen and enhance bacterial uptake by host cells.

In conclusion, we demonstrated that two fish CKs were released extracellularly during bacterial infection. The extracellular CsCKM-1/2 could bind to bacterial pathogens and promote bacterial phagocytosis by immune cells. Moreover, CsCKM-1/2 could function as DAMPs and activate antimicrobial immunity probably *via* interaction with TLR2 (**Figure 6**). These findings provided new insights into the immune function and regulation of CK in teleost.

## Data availability statement

The original contributions presented in the study are included in the article/**Supplementary Material**. Further inquiries can be directed to the corresponding authors.

## Ethics statement

The animal study was reviewed and approved by Ethics Committee of Institute of Oceanology, Chinese Academy of Sciences.

## Author contributions

SJ, LS, and XL designed the study; XL conducted the experiments, analyzed the data, and wrote the first draft of the manuscript; LS and SJ edited the manuscript. All authors contributed to the article and approved the submitted version.
