# Supplementary material for: Tongue sole creatine kinases function as DAMP and activate antimicrobial immunity via TLR2
Source: Front Immunol. 2023 Mar 1;14:1142488. doi: 10.3389/fimmu.2023.1142488 (PMC10014616; doi:10.3389/fimmu.2023.1142488)
Supplement: Supplementary file 1 [file DataSheet_1.docx]

Supplementary Material

**Tongue sole creatine kinases function as DAMP and activate antimicrobial immunity via TLR2**

**Xin Li^1,2,3^, Shuai Jiang^1,2,3*^, Li Sun^1,2,3*^**

***Correspondence:** Shuai Jiang, [sjiang@qdio.ac.cn](mailto:sjiang@qdio.ac.cn); Li Sun, [lsun@qdio.ac.cn](mailto:lsun@qdio.ac.cn)

**Supplementary Table 1.** Primers used in this study.

| Primer | Sequences (5′→ 3′) | Gene |
| --- | --- | --- |
| CsCKM-1-pET30a-F | TAAGAAGGAGATATACATATGGCAAAGAACTGTCATAACGAC | CsCKM-1 |
| CsCKM-1-pET30a-R | GTGGTGGTGGTGGTGCTCGAGCTTCTGGGCGGGGATCAT |  |
| CsCKM-2-pET30a-F | TAAGAAGGAGATATACATATGCCTTTCGGTAACACACACA | CsCKM-2 |
| CsCKM-2-pET30a-R | GTGGTGGTGGTGGTG CTCGAG CTTCTGGGCAGGGATCATG |  |
| RT-CsCKM-1-F | TGGAGAGCGCAGAGCTATTG | CsCKM-1 |
| RT-CsCKM-1-R | CGTCGGACATACCAGTCAGG |  |
| RT-CsCKM-2-F | CGAGCGCAGACTGATTGAGA | CsCKM-2 |
| RT-CsCKM-2-R | AGTCATGGACTTCAGGGGGT |  |
| RT-Actin-F | GCACGGTATTGTGACCAACTGG | Actin |
| RT-Actin-R | CAGGGGAGCCTCTGTGAGC |  |
| RT-TNF-α-F  RT-TNF-α-R  RT-IL-1β-F  RT-IL-1β-R  RT-IL6-F  RT-IL6-R  CsCKM-1-pmCherry-F | TGACCAACTCGGTGGAATGG  GGAACGACACCTGGCTGTAA  CCTTCCCACTTCCTGGCTAC  TGGCGTGTGCTGGCTTTTAT  TCCTGCGTGATGGCATAGTG  CTCATCAGGACGTCAGAGCC  TCAGATCTCGAGCTCAAGCTTATGGCAAAGAACTGTCATAACGAC | TNF-α  IL-1β  IL6  CsCKM-1 |
| CsCKM-1-pmCherry-R | CATGGTGGCGACCGGTGGATCCCGCTTCTGGGCGGGGATCAT |  |
| CsCKM-2-pmCherry-F | TCAGATCTCGAGCTCAAGCTTATGCCTTTCGGTAACACACACA | CsCKM-2 |
| CsCKM-2-pmCherry-R | CATGGTGGCGACCGGTGGATCCCGCTTCTGGGCAGGGATCATG |  |
| CsTLR2-pEGFP-F | CTCAGATCTCGAGCTCAAGCTTCGTCTCCAACACCCCCATCCA | CsTLR2 |
| CsTLR2-pEGFP-R | TTATCTAGATCCGGTGGATCCTTACAGTCTGATGTCACAGGACACTT |  |
| CsTLR2-Flag-F | GATGACGACGATAAGGAATTCTCTCCAACACCCCCATCCA | CsTLR2 |
| CsTLR2-Flag-R | AATTAATTAAGATCTGCTAGCTTACAGTCTGATGTCACAGGACACTT |  |
| CsCKM-1-Flag-F | GATGACGACGATAAGGAATTCATGGCAAAGAACTGTCATAACGAC | CsCKM-1 |
| CsCKM-1-Flag-R | AATTAATTAAGATCTGCTAGCTTACTTCTGGGCGGGGATC |  |
| CsCKM-2-Flag-F | GATGACGACGATAAGGAATTCATGCCTTTCGGTAACACACACA | CsCKM-2 |
| CsCKM-2-Flag-R | AATTAATTAAGATCTGCTAGCCTACTTCTGGGCAGGGATCATG |  |
| CsTLR2-Myc-F | TTCTGAAGAGGACTT GAATTCATCTCCAACACCCCCATCCA | CsTLR2 |
| CsTLR2-Myc-R | ACGACTCACTATAGTTCTAGATTACAGTCTGATGTCACAGGACACTT |  |
| CsCKM-1-Myc-F | TTCTGAAGAGGACTTGAATTCAATGGCAAAGAACTGTCATAACGAC | CsCKM-1 |
| CsCKM-1-Myc-R | ACGACTCACTATAGTTCTAGATTACTTCTGGGCGGGGATC |  |
| CsCKM-2-Myc-F | TTCTGAAGAGGACTTGAATTCAATGCCTTTCGGTAACACACACA | CsCKM-2 |
| CsCKM-2-Myc-R  RT-Hepcidin-F  RT-Hepcidin-R  RT-NK-lysin-F  RT-NK-lysin-R  RT- Saposin-F  RT-Saposin- R | ACGACTCACTATAGTTCTAGACTACTTCTGGGCAGGGATCATG  TGGATCAATGGATTACACCGT  CGCCGCTCAGTATTTACAACA  AAGCCTGCAACCCAAAGGAA  TCGTGCACACTTGAGGGTTT  CGGACACCTCACCACAAAGT  GACAGATCACTGCGTCCTCC | Hepcidin  NK-lysin  Saposin |


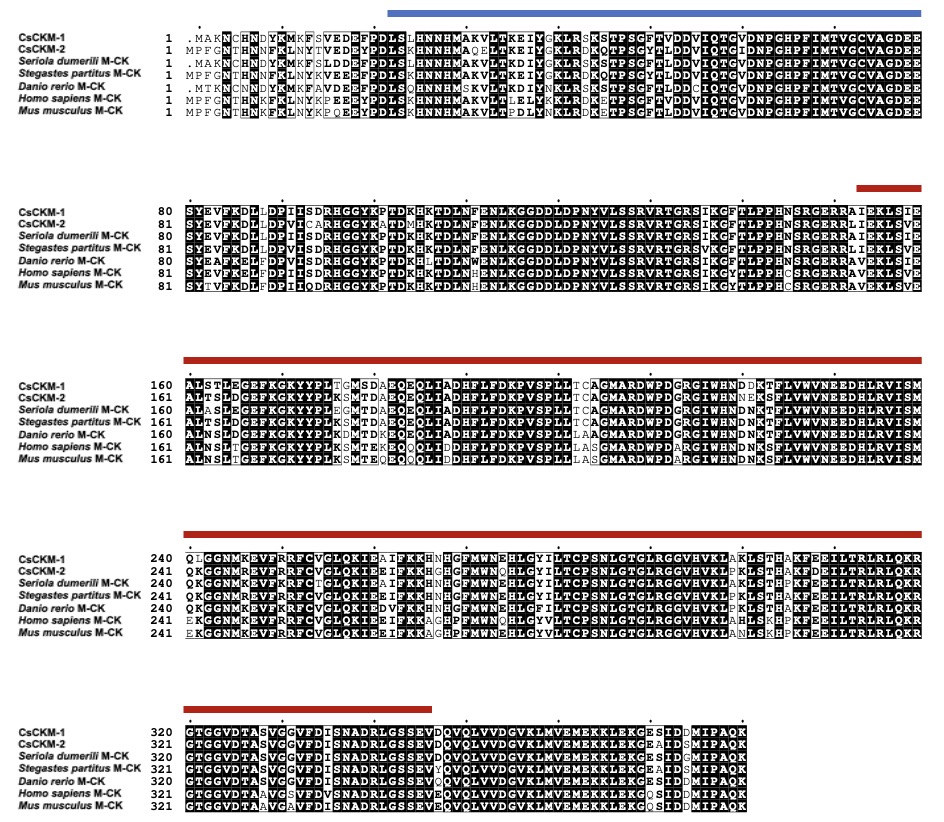


**Supplementary Figure 1**. Sequence alignment of CsCKM-1/2 with other CKs. The blue and red lines indicated the N- and C-terminal domains.


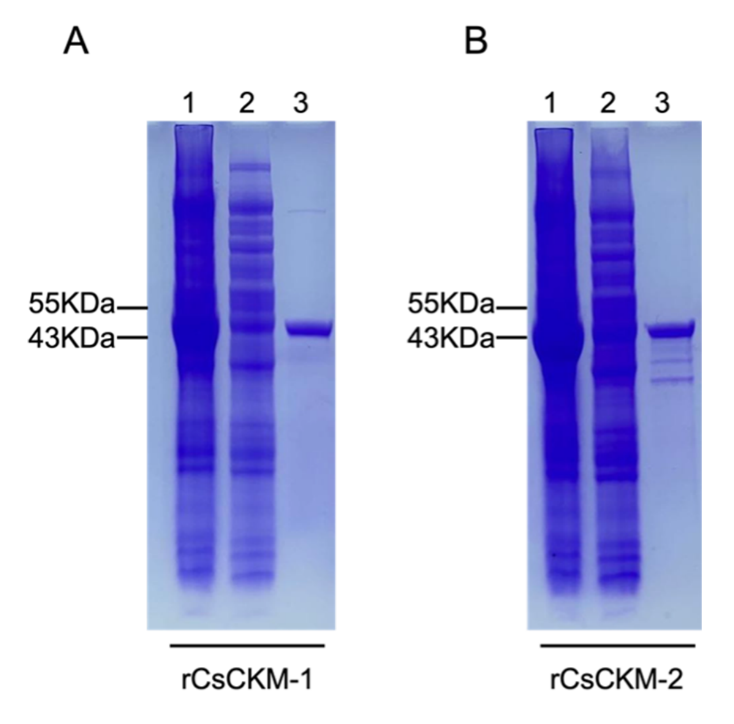


**Supplementary Figure 2**. Analysis of purified recombinant CsCKM-1/2. The supernatant of cell lysate (lane 1), flow through (lane 2), and purified rCsCKM-1 (**A**, lane 3) and rCsCKM-2 (**B**, lane 3) were analyzed by SDS-PAGE and viewed after staining with Coomassie brilliant blue R-250.


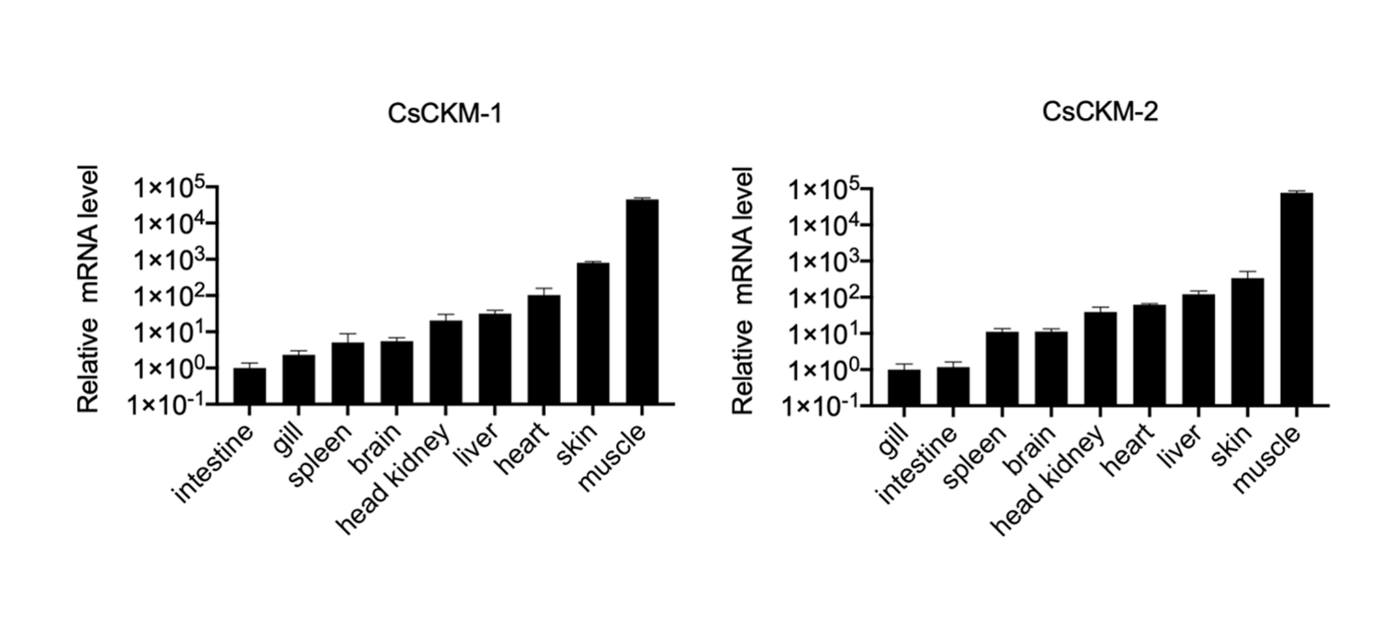


**Supplementary Figure 3.** The expression profiles of CsCKM-1/2 in tongue sole tissues as determined by qRT-PCR. The expression level in intestine (for CsCKM-1) or gill (for CsCKM-2) was set as 1.


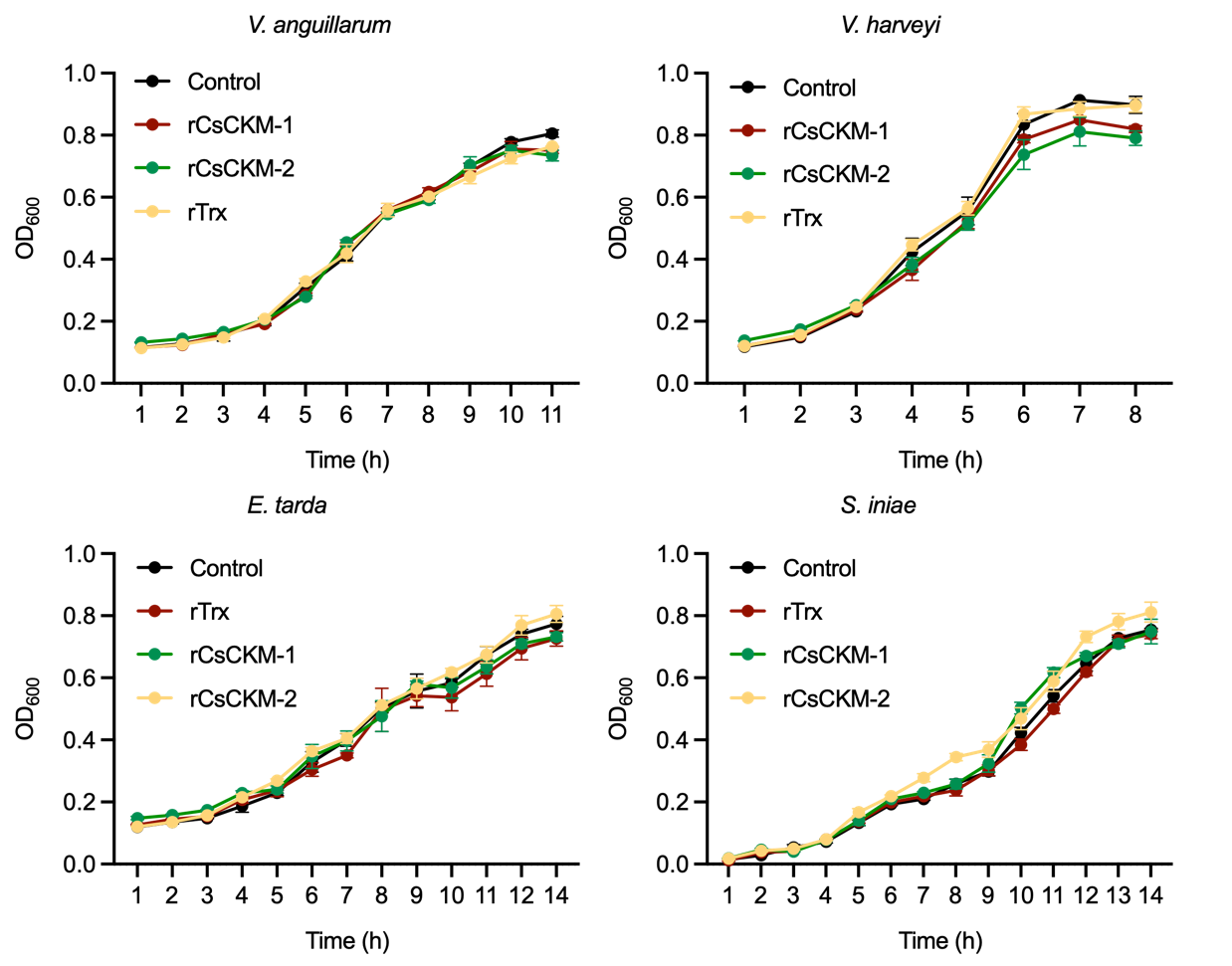


**Supplementary Figure 4.** The effect of CsCKM-1/2 on bacterial growth. *Vibrio anguillarum*, *Vibrio harveyi*, *Edwardsiella tarda* and *Streptococcus iniae* were incubated with or without (control) rCsCKM-1, rCsCKM-2, or rTrx at 22°C, the bacterial growth was monitored by measuring the absorbance at 600 nm.

**
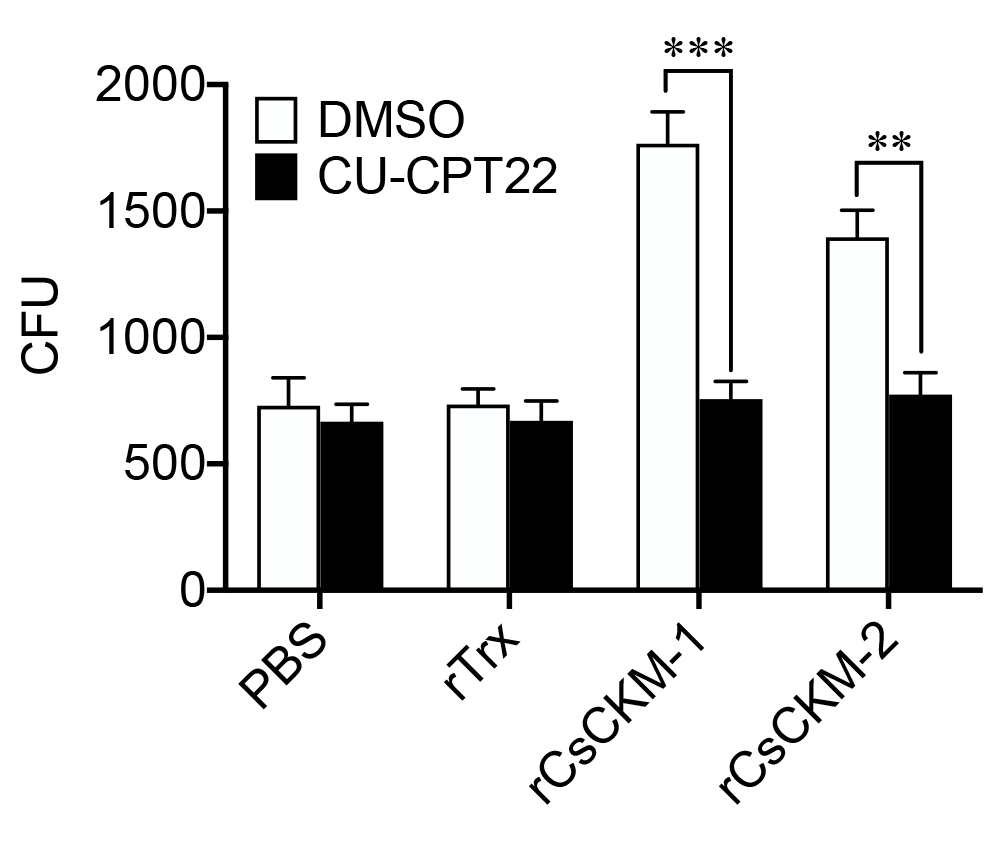
**

**Supplementary Figure 5.** The effect of CU-CPT22 on rCsCKM-1/2-induced phagocytosis. Tongue sole PBLs were preincubated with CU-CPT22 or DMSO (control), and then treated with rCsCKM-1/2, PBS or rTrx for 2 h. The cells were then incubated with *E. tarda* for 2 h. The number of intracellular bacteria (shown as colony forming unit, CFU) was determined by plate count. Data are expressed as the means ± SD, n = 3. **P < 0.01; ***P < 0.001.
